# Awareness of Losses and Not Gains Predicts Moderate-to-Vigorous Physical Activity

**DOI:** 10.1093/geroni/igaa057.952

**Published:** 2020-12-16

**Authors:** Erica O’Brien, Manfred Diehl, Eric Cerino, David Almeida

**Affiliations:** 1 Pennsylvania State University, University Park, Pennsylvania, United States; 2 Colorado State University, Fort Collins, Colorado, United States

## Abstract

The current study used data from a nationally representative sample of middle-aged and older adults to examine the synergistic relationship between negative and positive self-perceptions of aging on two types of physical activity (PA) and sedentary behavior. Participants from Wave 3 of the National Study of Daily Experiences reported on their awareness of age-related losses and gains, the frequency, duration, and intensity of their PA, and their sedentary behavior across seven days. We performed separate hierarchical models that regressed moderate-to-vigorous exercise, walking, and sitting on perceived losses and gains. Results revealed a significant main effect of perceived losses on moderate-to-vigorous exercise (p=.005, 95% CI=[-121.79,-22.267]). People who perceived more losses than average also reported engaging in fewer minutes of effortful PA. Results also suggested a marginal trend for more perceived losses to predict less walking (p=.055, 95% CI=[-42.85,0.42]) but to have no impact on sitting time (p=.58). Neither perceived gains nor the interaction between perceived gains and perceived losses emerged as significant predictors (ps>.17). Previous work demonstrates a facilitative role played by positive (self-)perceptions of aging on engagement in preventive health behaviors that have important impacts on long-term developmental outcomes. We explored additive and multiplicative effects associated with gain- and loss-related perceptions on PA and sedentary behavior to improve our understanding of the psychological context that surrounds the views of aging and health link. Initial findings suggest differential consequences of positive and negative self-perceptions of aging on distinct health-related behaviors, with the latter being stronger and contributing to activity inhibition.

